# Effect of Wu Qin Xi exercises on pain and function in people with knee osteoarthritis: A systematic review and meta-analysis

**DOI:** 10.3389/fmed.2022.979207

**Published:** 2022-11-07

**Authors:** Jiale Guo, Caiju Peng, Ziyan Hu, Liangliang Guo, Ru Dai, Yehai Li

**Affiliations:** Department of Orthopedics, Chaohu Hospital of Anhui Medical University, Hefei, China

**Keywords:** knee osteoarthritis, Wu Qin Xi, qigong, Chinese traditional medicine, pain, function, systematic review, meta-analysis

## Abstract

**Background:**

As a chronic disease that affects the whole world, there is no definite treatment for knee osteoarthritis (KOA). Wu Qin Xi (WQX) is still in preliminary exploration as a traditional Chinese exercise in the treatment of osteoarthritis of the knee. The purpose of this study was to conduct a meta-analysis of previous studies and to investigate the efficacy of the WQX exercises on pain and function in patients with KOA.

**Methods:**

We searched six databases (Pubmed, Embase, Cochrane Library, Wanfang, CQVIP, and CNKI) for articles on WQX for KOA up to May 10, 2022. Literature search, study selection, data extraction, and quality evaluation were performed by two independent authors. In terms of statistical results, we presented mean differences (MD), 95% CI, and *I*^2^ to show heterogeneity, and, based on that, we chose either a random effects model or a fixed effects model.

**Results:**

Seven studies were selected for inclusion in this meta-analysis. The WQX intervention group showed statistical differences for both the total Western Ontario and McMaster Universities Osteoarthritis Index (WOMAC) score and its various bylaws, the Visual Analogue Score (VAS), and the presence of general functional exercise in the control group. We also demonstrated the clinically meaningful efficacy of WQX treatment by calculating minimum clinical importance difference (MCID) values that met the MCID values on the WOMAC score. A sensitivity analysis was also performed in this study by subgroup analysis for greater heterogeneity, and it was inferred that the difference in follow-up time was a likely source of heterogeneity.

**Conclusion:**

Despite some limitations, the current study showed a definite effect of WQX in improving pain symptoms and joint function in patients with KOA.

**Systematic review registration:**

https://www.crd.york.ac.uk/prospero/, identifier: CRD42022332209.

## Introduction

As a chronic, universal, degenerative joint disease, knee osteoarthritis (KOA) has placed a heavy burden on sufferers, their families, and society (1). Along with discomfort, deformity, and the functional loss, KOA can increase the risk of cardiovascular events, venous thromboembolism, and fractures. An epidemiological study revealed that 60–70% of persons over 65 years are affected by KOA (2). Since KOA cannot be cured, pain relief and functional improvement are the main therapeutic objectives in treatment. Stepped therapy should be used as a guide in treating KOA. A customized treatment plan should be created based on the patient's age, gender, weight, risk factors, the severity of the lesion, and symptoms (3). Exercise is advised as the first line of treatment in most international standards, regardless of the severity of KOA. It is strongly advised for people with KOA to engage in low to moderate-intensity aerobic exercise and water-based activity (4, 5).

Trials were conducted to incorporate some traditional Chinese aerobic exercises into the treatment of KOA (6). Tai Chi and qigong are the most representative forms of exercise (7–10). Several high-quality randomized controlled trials (RCTs) have demonstrated its improvement in pain and physical function. Wu Qin Xi (WQX), an important part of the qigong exercise, was designed by Hua Tuo, a famous doctor of traditional Chinese medicine. Hua Tuo created WQX qigong by replicating the movements of five animals, including the tiger, bear, deer, ape, and crane, based on earlier studies (11, 12). The simplified WQX is currently being promoted in China, which is newly compiled by the Chinese Sports Committee. Each play contains two movements, which are done symmetrically once on each side and with breath conditioning, with the following actions: tiger standing up and lunging forward to eat; deer holding its horns and running; bear shaking its arms and swaying its body; ape lifting and picking things upwards; crane stretching and flying. There are many movements that may benefit the knee joint, for example, the support and weight shift of the knee joint in a semi-squat position in the tiger and deer movements, and the dynamic flexion and extension of the knee in a single-leg support position in the bird movement. In addition to physical exercise, WQX includes some Chinese medical philosophies (such as “Qi”), which are not found in other common functional exercises (13). According to traditional Chinese medicine, WQX can restore the balance of yin and yang in the human body by properly stretching our body with specific breathing patterns coupled with the intentional movement of heavenly yang and earthly yin. Qi is the essence of life and determines whether all life on earth and in heaven exists in traditional Chinese culture (14). WQX qigong places a strong emphasis on using Qi while conditioning the body through movement and breathing (15). Based on this traditional Chinese medicine theory and the observation that the WQX movements are indeed beneficial to the functional exercise of the knee joint, people started experimenting with the use of WQX in the fundamental treatment of KOA. We wanted to explore if a meta-analysis of prior studies could conclude that the WQX exercise is beneficial for pain symptoms and joint function in KOA.

## Methods

### Study protocol and registration

All analyses were based on data from previously published studies. Consequently, no ethical approval or patient consent was required. This systematic review and meta-analysis were conducted according to the Preferred Reporting Items for Systematic Reviews and Meta-Analyses (PRISMA) guidelines (16). An a priori protocol for the review is published in the International Prospective Register of Systematic Reviews (PROSPERO): CRD42022332209.

### Search strategy and study selection

Two independent reviewers (Hu and Peng) searched Pubmed, Embase, Cochrane Library, Wanfang, CQVIP, and CNKI databases to identify relevant studies up to 10 May 2022. In Pubmed, we employed this search strategy: ((“Osteoarthritis, Knee”[Mesh]) OR ((((Knee Osteoarthritides[Title/Abstract]) OR (Knee Osteoarthritis[Title/Abstract])) OR (Osteoarthritis of Knee[Title/Abstract])) OR (Osteoarthritis of the Knee[Title/Abstract]))) AND (((((wuqinxi[Title/Abstract]) OR (wu qin xi[Title/Abstract])) OR (WQX[Title/Abstract])) OR (wuqinxi qigong[Title/Abstract])) OR (five animal exercise[Title/Abstract])). We use a similar search methodology in other databases as well. The criteria for inclusion from the literature were as follows: (I) All of the included literature contained relevant clinical trials. (II) All participants in the included studies were diagnosed with KOA at a tertiary care hospital and with knee pain symptoms (17–19). (III) WQX exercise was compared to a blank control or another functional exercise in trials. (IV) The outcome metrics included are the Western Ontario and McMaster Universities Osteoarthritis Index (WOMAC) or the Visual Analogue Score (VAS) (20, 21). (V) No restrictions in the language of the article.

### Data extraction and quality assessment

Data extraction and quality assessment from original articles were performed independently by two authors (Hu and Peng). When any information was unclear, we attempted to contact the relevant author of the original article. For data extraction and transformation, the differences were selected by a senior director (Li) with the reference to the original literature. The primary outcome indicator for our meta-analysis was WOMAC, and the secondary outcome indicator was VAS. WOMAC is a self-evaluation scale widely used to assess the severity and treatment efficacy of hip and knee conditions in three major areas: pain, stiffness, and joint function. It has a total of 24 items, including five items for pain assessment, two items for stiffness, and 17 items for joint function. The total score is the sum of the 24 items, with a higher total score representing a more severe disease. WOMAC is available in two formats: visual analog scale and Likert scale, both of which have similar metric properties. If the units of the WOMAC scores differed between the included studies, we translated them into results based on the 100 mm VAS evaluation scale (total score of 2,400, total pain score of 500, total stiffness score of 200, and total joint function score of 1,700). The VAS is the most commonly used unit measure of pain intensity. The scale consists of a 100 mm straight line with one end of the line indicating “no pain at all” and the other end indicating “extreme pain.” Patients were asked to mark the appropriate location on the line to represent the intensity of pain they are experiencing at that time. The smaller the value of the measurement, the less painful it is, with 0 being no pain and 10 being the most painful. The extracted items included the following: (1) author, (2) time of publication; (3) study location; (4) sample size; (5) mean age; (6) KOA duration; (7) grade of KOA; (8) interventions; (9) controls; (10) duration time; (11) follow-up time; (12) outcome measure. Cochrane Risk of Bias Assessment tool (22) (including seven items about methods, assessment, reporting, and the other bias) was used by two independent reviewers (Hu and Peng) to assess the quality of included literature. Each item was scored as high risk, uncertain risk, or low risk. For some items that we scored as uncertain risks, we contacted the author and requested data for further analysis. All the controversies were resolved following a third reviewer's (Li) opinion.

### Statistical analysis

The outcome indexes we contracted from the original article were continuous data. Based on the guidance of the Cochrane handbook (23), outcome data were used in the meta-analysis, in the absence of baseline characteristics without differences. The mean difference (MD) (rather than standardized mean difference) and 95% confidence interval (CI) in outcomes were considered as effect indexes since we onlv included the same evaluation scale in the same forest plot. The decision of the included studies on which statistical model (fixed-effect model or random-effect model) to analyze the data depends on the significant heterogeneity between studies. To assess heterogeneity, we used the *I*^2^ statistics: *I*^2^ represented the size of heterogeneity while *I*^2^ ≤ 50% was defined as acceptable in the Cochrane handbook. The fixed-effect model was chosen if *I*^2^ ≤ 50%; otherwise, the random-effect model was used. If there was significant heterogeneity among the results, we conducted a sensitivity analysis to find the source of heterogeneity by using subgroup analysis. If the *p*-value is < 0.05, it indicates a statistical significance. Meta-analysis was performed using RevMan 5.4 software, and all data analysis was shown in the form of forest plots.

Furthermore, we calculated the minimum clinical importance difference (24) (MCID) value for WOMAC and VAS. The *p*-values are typically utilized to make decisions regarding the interpretation of scale data. An increasing number of academics are concluding that it is not scientific to evaluate efficacy solely based on the *p*-value of the hypothesis test for the difference between pre- and post-treatment scale scores and that a statistically significant *p*-value does not necessarily imply that it is clinically significant. Therefore, the *p*-values alone should not be the only factor in clinical research, and the size of the *p*-values alone does not represent the size of clinical differences. In this regard, the MCID was introduced to determine whether there is a clinically significant difference. The MCID value was improved by more than 12% from baseline scores in WOMAC (25) and 1.8 units in VAS (26).

## Results

### Research selection

**Figure 1:**
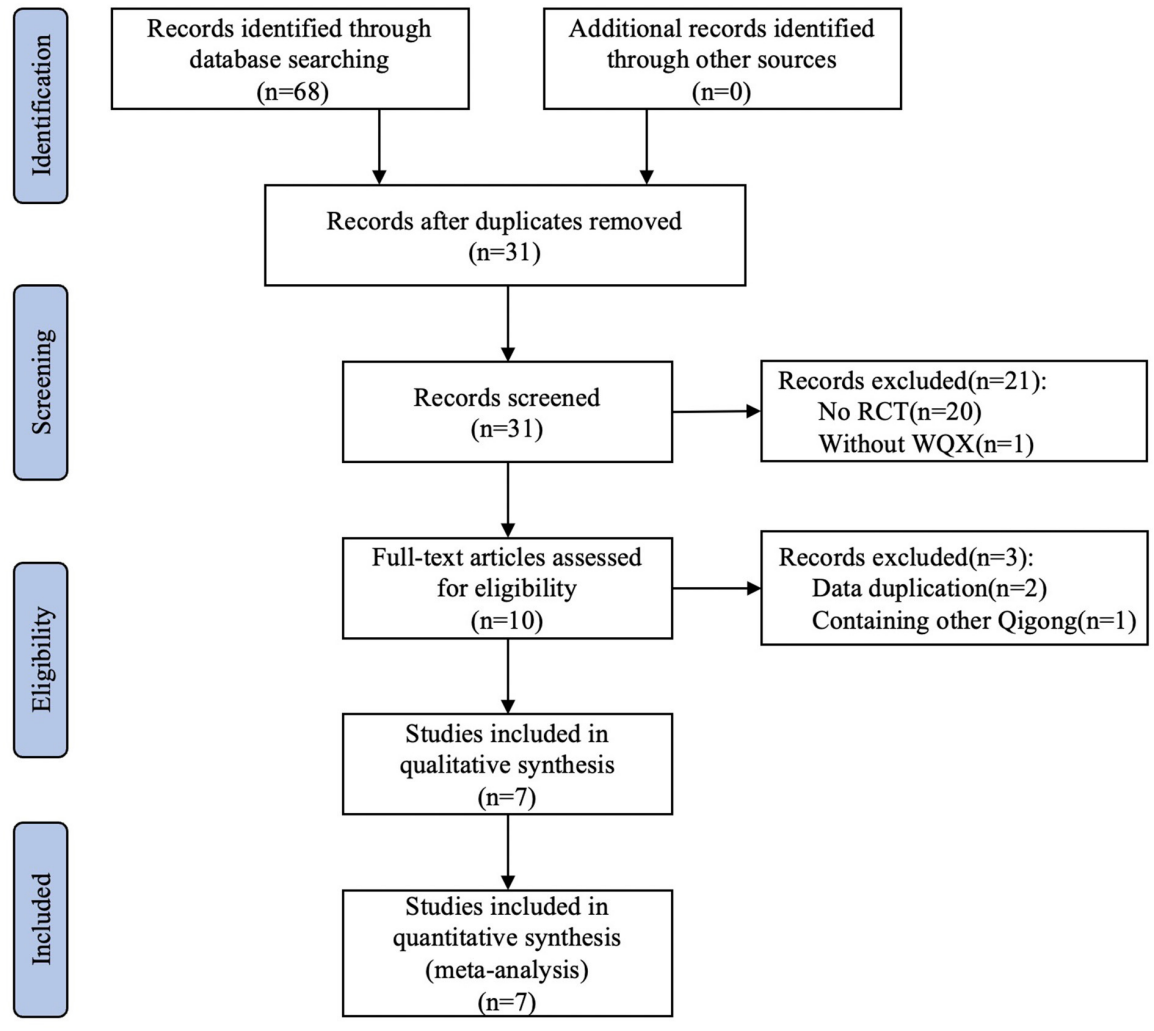
Flowchart of the trial selection process.

Through a literature search, we retrieved 68 relevant papers from six databases. After eliminating duplicates, there were remaining 31 papers (Figure 1). From the remaining 31 papers, we excluded 20 papers after coarse screening because they did not contain RCTs, and one paper was excluded since it did not contain WQX. After screening the remaining 10 papers, we found that two of them had the same authors and units as the other two by reading the full text. Following careful evaluation, despite the difference in sample size and participant characteristics, the likelihood of duplication in the test population of these papers remained higher after our comprehensive judgment, thus we excluded two of them. An additional paper was excluded since the control group included other traditional Chinese sports. Finally, seven studies met the inclusion criteria (27–33).

### Characteristics of included study

All the references were published within 10 years (2012–2021). All the trials were conducted in different parts of China, and participants were Chinese (100%) without mentions of ethnicity. The total sample sizes were 668 participants; of which, 338 were in the experiment group and 330 were in the control group. Most of the included subjects were diagnosed with KOA with chronic pain over 6 months in tertiary hospitals and were older than 50 years. In total, only five trials reported KOA classification, and all were based on the Kellgren-Lawrence scale. Three of these trials had participants in Class I/II and two studies in Class II/III. The intervention was the WQX exercise in all studies while one study included Tui Na (a Chinese physical treatment) and isokinetic training. The control groups were either blank control or the other physical treatment. The duration of trials ranged between 3 and 6 months, such is the case for follow-up time. WOMAC and VAS were included as outcome measures in this meta-analysis, where six references reported WOMAC and two references reported VAS. Three of the references for WOMAC were based on the VAS 100 mm format (total score: 2,400), two were based on the Likert scale (0–4) format (total score: 96), and one was based on the VAS 10 mm format (total score: 240). We all transformed them under the same evaluation scale for comparison. The main characteristics of extracted study are shown in Table 1.

**Table 1:** Characteristics of data extracted from the included studies.

**References**	**Study location**	**Participant characteristics**				**Intervention protocol**				**Outcome measure**
		**Sample size**	**Mean age (year)**	**KOA duration (month)**	**Grade of KOA (K/L scale)**	**Intervention group**	**Control group**	**Duration time**	**Follow-up**	
Li et al. (27)	Fujian	55/53	EG:58.51 ± 1.20	EG:17.64 ± 1.13	II/III	WQX + TuiNa	TuiNa + isokinetic	20 days (WQX	6 months	VAS
	China		CG:57.09 ± 1.22	CG:19.26 ± 1.23		+ isokinetic training	training	6 months)		
Tian et al. (28)	Sichuan	20/20	EG:63.0 ± 4.0	EG/CG≥6	I/II	WQX	None	6 months	6 months	WOMAC
	China		CG:62.0 ± 3.9							
Tu and Liao (29)	Sichuan China	20/20	EG/CG≥50	EG/CG≥6	I/II	WQX	Standing exercise	16 weeks	16 weeks	WOMAC
Wang et al. (30)	Tianjin	18/10	EG:65.00 ± 5.18	EG:5.31 ± 4.31	II/III	WQX	None	12 weeks	12 weeks	VAS;
	China		CG:66.20 ± 5.33	CG:5.27 ± 3.07						WOMAC
Xiao et al. (31)	Beijing	34/34	EG:70.7 ± 9.36	EG:12.21 ± 7.38	I/II	WQX	Physical therapy	12 weeks	3 months	WOMAC
	China		CG:70.2 ± 10.35	CG:12.81 ± 5.24						
Xiao et al. (32)	Hubei	132/134	EG:71 ± 2.92	EG:28.3 ± 18.10	None	WQX	None	24 weeks	24 weeks	WOMAC
	China		CG:69 ± 3.72	CG:27.9 ± 17.98						
Yin and Li (33)	Anhui	59/59	EG:68.6 ± 2.3	None	None	WQX	None	3 months	3 months	WOMAC
	China		CG:69.6 ± 2.3							

K/L scale, Kellgren-Lawrence scale.

### Assessment of risk of bias

**Figure 2:**
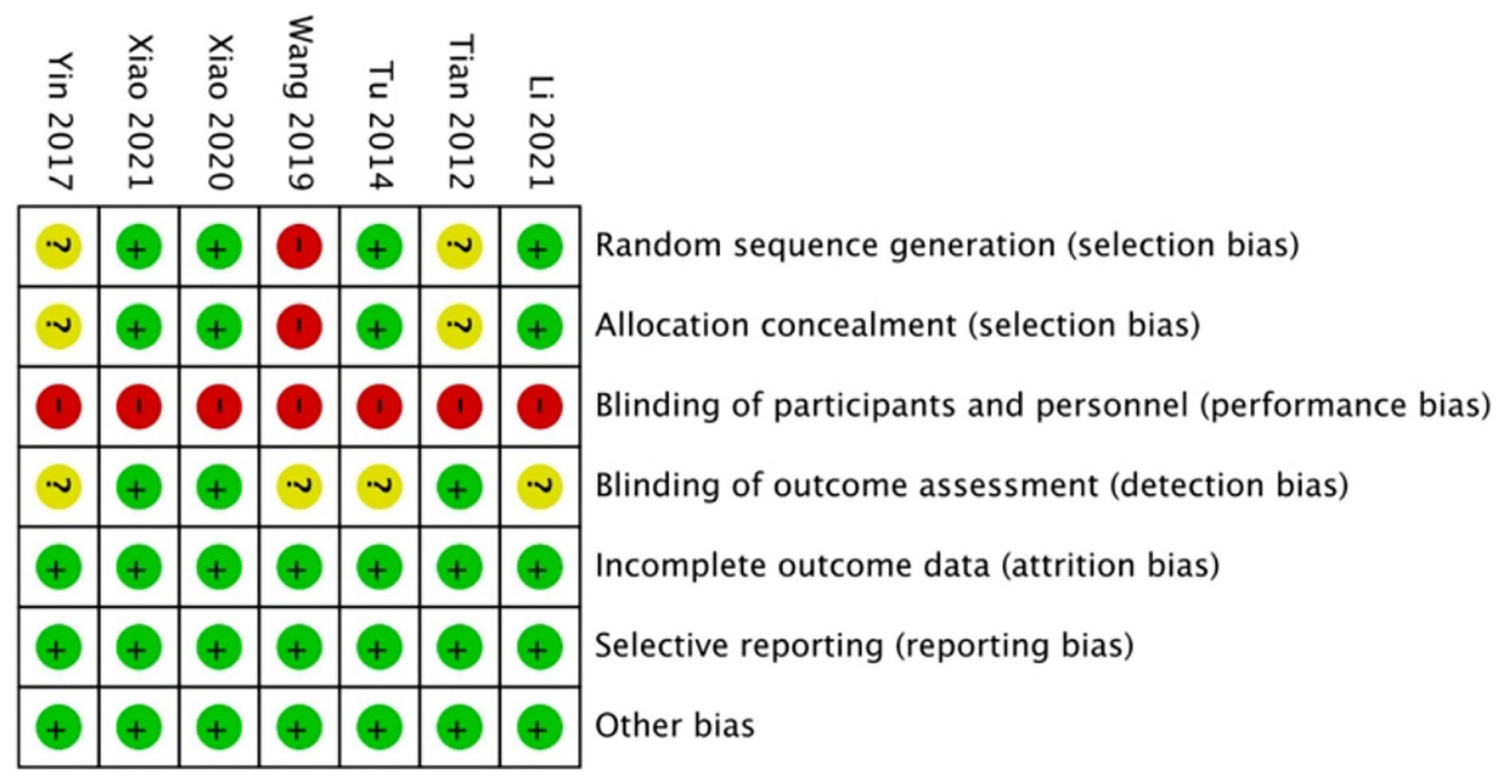
Risk of bias summary: review authors' judgments about each risk of bias item for each included study.

The results of the quality assessment are shown in Figure 2. Four trials provided some information about the appropriate method of randomization and the selection bias was avoided. One trial allocation method was not reported while two did not report the method used to generate and conceal the allocation sequence. In the performance bias section, all trials were judged to be at high risk due to the inevitability of participants knowing whether they performed the WQX exercise or not. For detection bias, three trials showed that there were independent reviews while the others did not provide this information. Detection bias, attrition bias, and reporting bias were evaluated as low risks from the original article.

### Outcomes of meta-analysis

**Figure 3:**
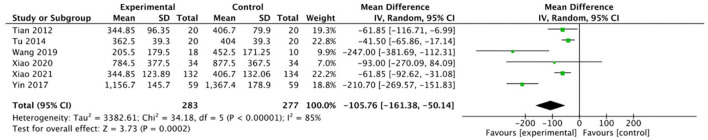
WOMAC total score.

**Figure 4:**
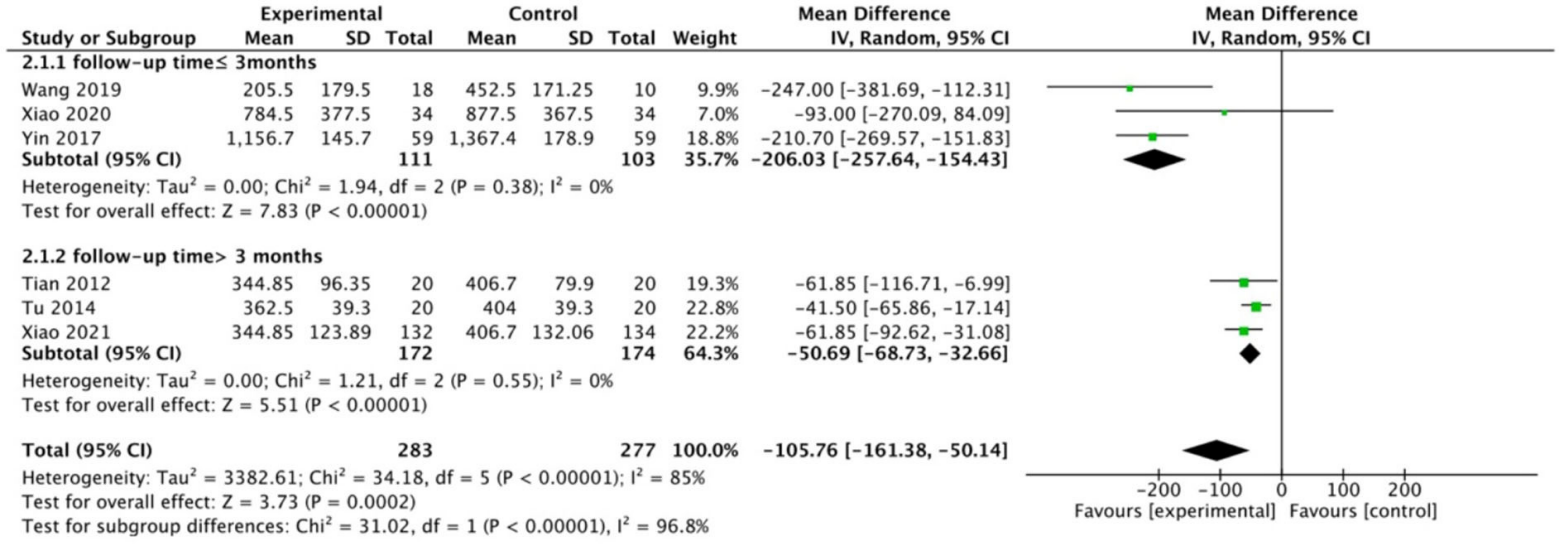
Subgroup analysis by follow-up time.

The Western Ontario and McMaster Universities Osteoarthritis Index total score was reported in six studies. This meta-analysis indicated that the WQX exercise has a significant improvement in WOMAC total score regardless of the intervention of control group (MD = −105.76; 95% CI: −161.38 to −50.14; *p* < 0.01, *I*^2^ = 85%, Figure 3). However, it indicated that there was strong heterogeneity across these studies. To address this issue, we performed a subgroup analysis. We classified six studies into two subgroups by follow-up time (Figure 4). When subgroup analyses were performed, we found that the heterogeneity disappeared (follow-up time ≤ 3 months: MD = −206.03; 95% CI: −257.64 to −154.43; *p* < 0.00001, *I*^2^ = 0%; follow-up time ≥ 3 months: MD = −50.69; 95% CI: −68.73 to −32.66; *p* < 0.00001, *I*^2^ = 0%; total: MD = −105.76; 95% CI: −161.38 to −50.14; *p* = 0.0002, *I*^2^ = 85%; test for subgroup difference: *I*^2^ = 96.8%). It showed that the timing of follow-up has contributed to the heterogeneity. For this reason, when we analyzed different control group interventions, we also performed subgroup analysis on the follow-up period. Furthermore, for WOMAC, we calculated an improvement of 16.2%>12% (MCID) relative to baseline values, with a clinical difference. In WOMAC scores, WQX was therefore not only statistically different but also clinically significant.

**Figure 5:**
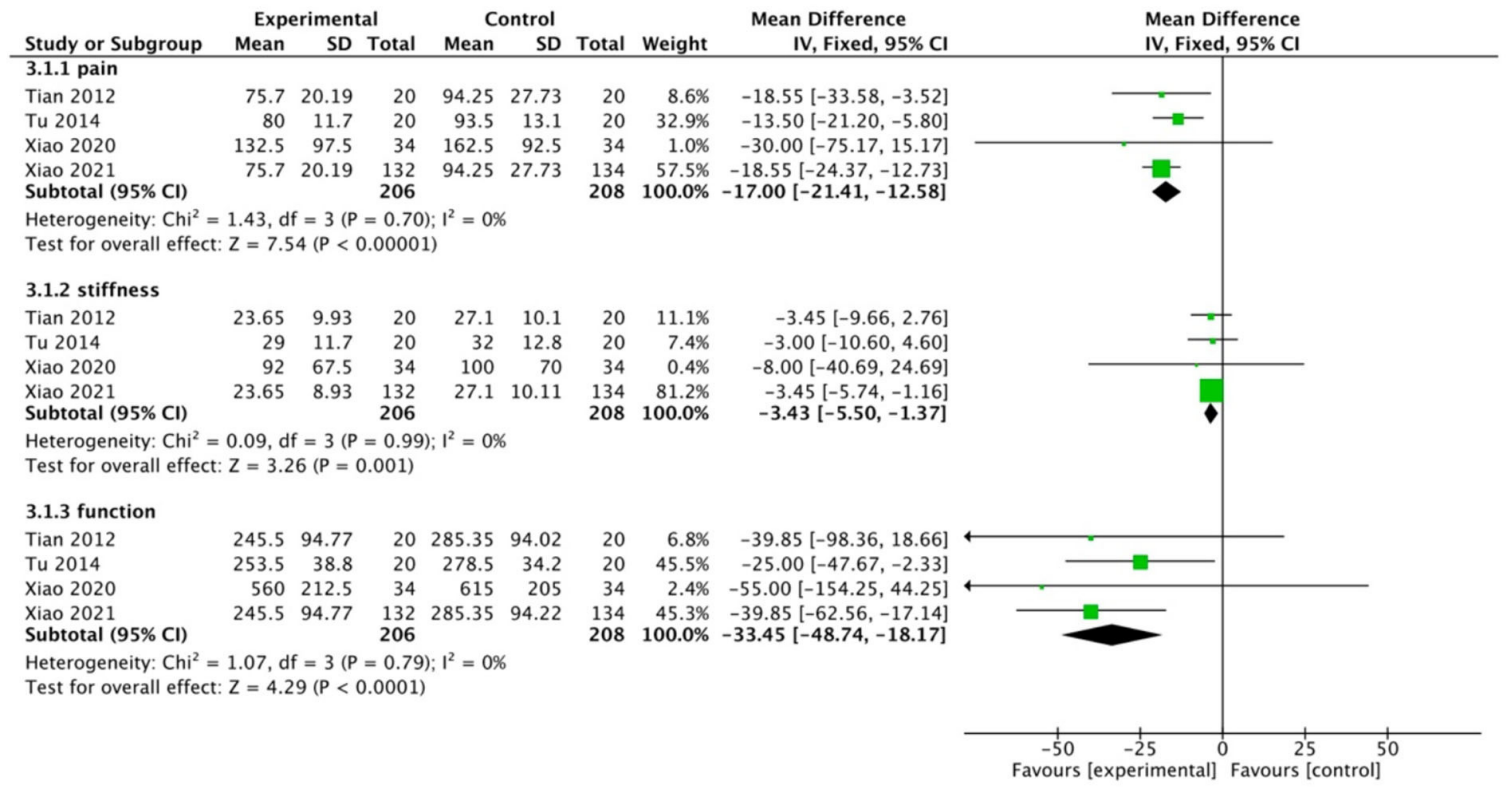
WOMAC-based pain, stiffness, and functional ratings.

Then, we ran separate analyses of trials using WOMAC-based pain, stiffness, and functional ratings. In total, four studies involving 414 participants reported these indicators, and we put these analyses in the same forest plot, as shown in Figure 5. The WQX exercise significantly improved the pain symptoms (MD, −17.00; 95% CI: −21.41 to −12.58; *p* < 0.00001, *I*^2^ = 0%), joint stiffness (MD, −3.43; 95% CI: −5.50 to −1.37; *p* = 0.001, *I*^2^ = 0%), and joint function (MD, −33.45; 95% CI: −48.74 to −18.17; *p* < 0.0001, *I*^2^ = 0%) of participants.

**Figure 6:**
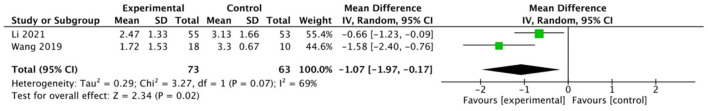
VAS.

Our examination of the two studies that reported VAS scores revealed that there was an improvement (MD, −1.07; 95% CI: −1.97 to −0.17; *p* = 0.02, *I*^2^ = 69%, Figure 6). From the previous analysis, we can speculate that this heterogeneity may be due to differences in the duration of follow-up (The follow-up period for one research was 6 months, while the other was 3 months). We did not do a sensitivity analysis in this case since there were only two trials (34). In the case of VAS, the results of the meta-analysis showed an improvement of only 1.07 < 1.80 (MCID) relative to the control group, which to some extent suggested that there was no clinical significance. We need to be cautious about the results of the meta-analysis in terms of VAS.

**Figure 7:**
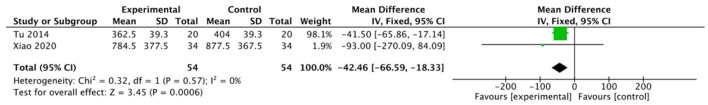
Control group with other functional exercises.

**Figure 8:**
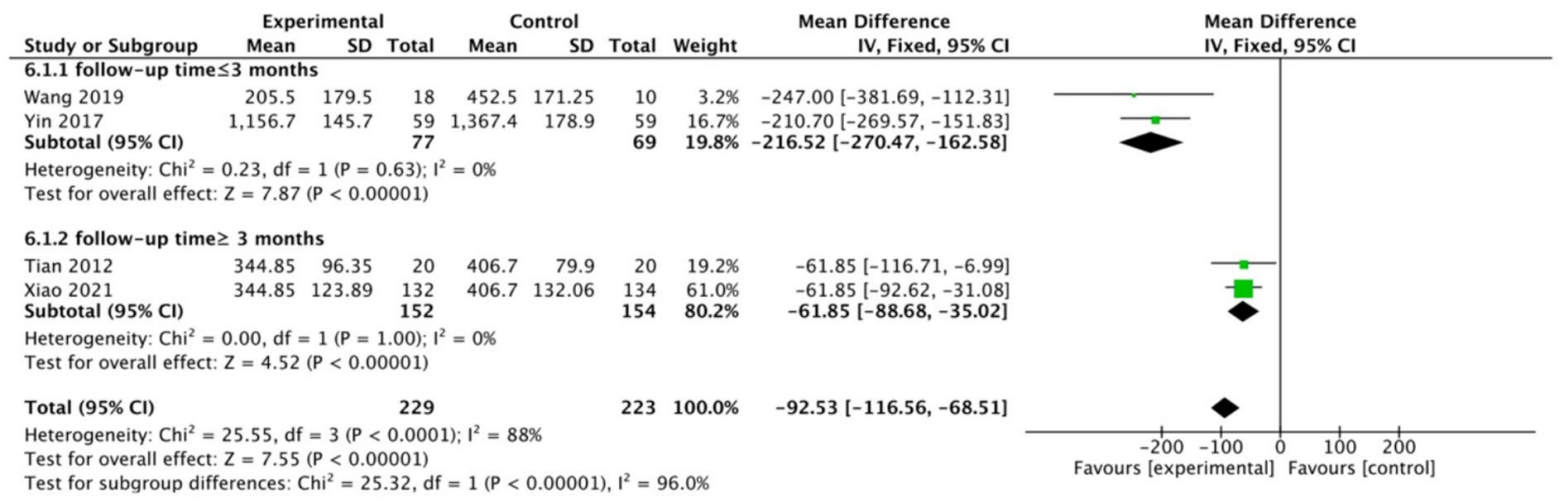
Control group without intervention.

We grouped the controls according to the existence of other exercise modalities and analyzed them separately. The results showed that WQX exercise showed a statistical difference regardless of whether the control group had other functional exercises or not. When analyzing the trials compared to the control group with other functional exercises, the combined results showed that WQX was effective compared to other common functional exercises (MD: −42.46; 95%CI: −66.59 to −18.33; *P* = 0.0006, I^2^ =0%, Figure 7). Similarly, the integration analysis for the blank control group showed a statistical difference in the WQX exercise intervention (follow-up time ≤3 months: MD = −42.46; 95% CI: −66.59 to −18.33; *p* < 0.00001, *I*^2^ = 0%; follow-up time ≥3 months: MD = −61.85; 95% CI: −88.68 to −35.02; *p* < 0.00001, *I*^2^ = 0%; total: MD = −92.53; 95% CI: −126.56 to −68.51; *p* < 0.0001, *I*^2^ = 88%; test for subgroup difference: *I*^2^ = 96.0%, Figure 8). This subgroup analysis again demonstrated that differences in follow-up time were the source of heterogeneity.

## Discussion

The goal of this meta-analysis was to evaluate the effects of the WQX exercise on pain and function in people with KOA. Our meta-analysis was the first to evaluate the WQX exercise for KOA and found that the WQX exercise is generally effective in patients with KOA (*p* < 0.01). The MCID value also demonstrated the exact clinical significance of WQX in knee treatment. We found that the WQX exercise showed better efficacy compared with other conventional exercises (*p* < 0.01). Moreover, the duration of follow-up may be associated with the symptoms of the participants. In contrast to what we conceived, the subgroup analysis with a follow-up time not exceeding 3 months showed better effects than the subgroup with a longer follow-up. In general, the follow-up results of the WQX exercise should be better for longer workouts than for shorter ones. As we read through the original text of these studies, we noted that none of the studies reported problems with participant adherence or standardization of movements. These are factors that influence the efficacy of the WQX exercise. Since a WQX session takes about 60 min, the motivation of patients to participate is in fact an important problem as time passes. This could be the reason for this outcome.

For the analysis of VAS, although the combined results we obtained were statistically significant, MCID calculations showed that these results were not clinically significant. Moreover, the combined results were highly heterogeneous. Since the meta-analysis included only two studies, we could not find the source of heterogeneity by excluding the literature one by one, subgroup analysis, meta-regression, etc. By comparing the PICOS characteristics of the two included studies, we found that there were many differences in the trials. Li had a 3-week exercise program and was followed up at 6 months, whereas Wang had a 12-week exercise program and was followed up at 12 weeks. An integrated intervention protocol was used in Li's study: the test group consisted of WQX, TuiNa, and isometric muscle training, and the control group consisted of TuiNa and isometric muscle training. As a form of physical therapy, TuiNa is used to relieve pain by loosening soft tissues, such as muscles and increasing blood circulation, which often only provides short-term pain relief. The study only included pushing every 2 days for the first 3 weeks, which had essentially no effect on the 6-month follow-up results that were included in our analysis. However, isometric plyometric exercise, a low to moderate-intensity aerobic exercise, has been shown to enhance lower extremity muscle strength and provide some degree of improvement in knee pain and function. Although the difference between the two groups was only the presence or absence of WQX, this may also influence the evaluation of the efficacy of the WQX exercise to some extent. However, in our meta-analysis, Li's study only had VAS outcomes included in the analysis; therefore, the combined interventions in this study may have influenced the evaluation of WQX efficacy, which did not have an impact on the primary outcome (WOMAC) of our meta-analysis. Thus, this suggested that the results of the VAS meta-analysis need to be viewed with more caution.

This meta-analysis preliminarily demonstrated that the WQX exercise is an available alternative therapy for patients with KOA. WQX was demonstrated to be beneficial in reducing joint discomfort and joint stiffness (*P* < 0.01) and enhancing joint function (*P* < 0.01).

The pathophysiological mechanisms of osteoarthritis remain unclear at this stage. Previous studies have shown that the muscle strength of the quadriceps and hamstrings is significantly reduced in patients with KOA, with a more pronounced reduction in quadriceps muscle strength, which is associated with joint swelling and pain and impaired mobility (35). As a result, exercise therapy based on functional quadriceps exercises are recommended as the first line of basic treatment in many guidelines (36). WQX, a type of qigong that imitates animals, is designed to inspire people to learn when compared to other general functional exercises. The belief of elderly patients in traditional gong practices, particularly in China, allows them to continue with the exercise. In addition, the WQX exercise has become a popular form of fitness in China due to the emphasis on health and wellness. There are many knee joint flexion and extension, rotation, and weight shifting movements in the WQX exercise, for example, the crane exercise can strengthen the quadriceps. Therefore, in theory, the WQX exercise is effective in the treatment of knee patients, and this meta-analysis is an attempt to summarize previous studies done on that subject. There are studies and meta-analyses that show the precise effects of WQX in coronary heart disease, hypertension, low back pain, and osteoporosis, thus the content of this meta-analysis can widen the impact of WQX workouts on functional improvement in the elderly (37–40). It is also an attempt to apply traditional Chinese medical theory to modern scientific medicine.

There will undoubtedly be comparisons made between WQX and Tai Chi and other forms of qigong in China. Different gong methods have their own advantages and disadvantages, and their focus on improving the body's functions is also different. According to some studies, WQX is more effective than other gong techniques, including Tai Chi, in helping middle-aged and elderly people improve their lung function (41) and reduce oxidative stress (42). However, Tai Chi is more effective than WQX in treating hypertension (11). Qigong emphasizes the use of “Qi” and the balance of “Yin” and “Yang” in the body through the combination of breathing. In addition to the elements of general qigong, WQX has more characteristics in the use of ideas. In qigong methods, one idea is used from the beginning till the end, but in WQX, different ideas are applied for each scene. This kind of deliberate exercise can aid in the transformation and regulation of the mental, emotional, and psychological state of a person, assisting in the release of mental tension, the reduction of psychological stress, and the maintenance of a mental health state. WQX is still most frequently contrasted with Tai Chi as a traditional gong technique. WQX is much less well-known than Tai Chi both internationally and in China. As a result, people are even more motivated to learn Tai Chi. However, even though the version of Tai Chi is simplified, it is still more challenging to learn than WQX because it has 24 sets of movements as opposed to only 10 sets of movements in WQX. Furthermore, Tai Chi contains movements with extreme knee flexion, which should be very detrimental to the knee joint. In contrast, all movements in WQX movements have knee flexion no >90°. Therefore, although there is no RCT comparing WQX with Tai Chi in the treatment of osteoarthritis of the knee, we hypothesize that WQX is more suitable than Tai Chi for patients with KOA based on the analysis of movements.

This meta-analysis, however, has some limitations. The first and most serious limitation is that the number of trials included and the sample sizes are both too small. Although we thoroughly searched the six databases, there are databases in other languages, such as Japanese and Korean databases, that were not searched due to the language restrictions and insufficient experience of the authors. Because of the relatively large learning cost of WQX, especially for the learning ability of the elderly, it is difficult to conduct large-scale controlled trials, thus the sample size of clinical trials on WQX qigong is generally small. In addition, compared to Tai Chi, WQX qigong was promoted with less fervor and scope, which also influenced researchers' attention to the WQX movement to some extent. The second limitation is that, due to the specificity of the trial, blinding of patients is not possible, which may have some impact on the implementation of our RCTs. There were also some studies that did not provide enough information for us to evaluate the quality of the literature, which also influenced us in this meta-analysis. The third limitation is that no adverse events were reported in any of the included trials. Although the likelihood of adverse events is low, the safety of the WQX exercise is still not scientifically proven, thus there may be some bias. The fourth limitation is that all of the included trials had a short follow-up period. We observed that the follow-up time for all included trials did not exceed 6 months for the outcome indicators. The efficacy of functional exercise in KOA should require a longer follow-up period to produce more reliable results. The fifth limitation is that, for traditional Chinese exercises, such as WQX, standard movements are essential to achieve a therapeutic effect. Although there are studies in the included literature that reported exercising under professional supervision, neither the standardization of the participants' movements nor the participants' compliance (i.e., whether the purpose of the exercise at the time of the trial design was achieved) was reported. The sixth limitation is that the KOA class characteristics of individuals with KOA are heterogeneous, and the intensity and frequency of interventions vary. These differences may contribute to the appearance of bias.

Overall, this meta-analysis found that the WQX exercise was clinically and statistically significant in the treatment of patients with KOA. Despite the limitations of the current study, both in terms of the quality and number of trials, the efficacy of WQX is positive. The current study is only a preliminary exploration of the application of the WQX exercise to the treatment of KOA, and future studies should pay attention to improving the scientific nature of trial design and pursuing high-quality RCTs. Also, adverse events in patients need to be reported for others to objectively assess the safety of the WQX exercise.

## Data availability statement

The original contributions presented in the study are included in the article/supplementary material, further inquiries can be directed to the corresponding author.

## Author contributions

JG: conceptualization, writing—original draft preparation, and writing—review and editing. JG, CP, and ZH: methodology, software, formal analysis, and investigation. CP and ZH: validation and resources. YL, CP, and ZH: data curation. JG and YL: visualization and project administration. YL and RD: supervision. RD and LG: review and editing. RD: manuscript proof-reading. All authors have read and agreed to the published version of the manuscript.

## Conflict of interest

The authors declare that the research was conducted in the absence of any commercial or financial relationships that could be construed as a potential conflict of interest.

## Publisher's note

All claims expressed in this article are solely those of the authors and do not necessarily represent those of their affiliated organizations, or those of the publisher, the editors and the reviewers. Any product that may be evaluated in this article, or claim that may be made by its manufacturer, is not guaranteed or endorsed by the publisher.
